# Development of a Polymerase Spiral Reaction-Based Isothermal Assay for Rapid Identification of *Thrips palmi*

**DOI:** 10.3389/fmolb.2022.853339

**Published:** 2022-05-02

**Authors:** Sumit Jangra, Amalendu Ghosh, Sunil Mukherjee, Virendra Kumar Baranwal, Ralf G. Dietzgen

**Affiliations:** ^1^ Insect Vector Laboratory, Advanced Centre for Plant Virology, ICAR-Indian Agricultural Research Institute, New Delhi, India; ^2^ Queensland Alliance for Agriculture and Food Innovation, The University of Queensland, Saint Lucia, QLD, Australia

**Keywords:** PSR, melon thrips, diagnostics, on-site detection, plant biosecurity, low-cost technique

## Abstract

Thrips cause considerable economic losses to a wide range of food, feed, and forest crops. They also transmit several plant viruses. Being cryptic, it is often difficult to distinguish thrips species in crops and large consignments by conventional methods. Melon thrips (*Thrips palmi* Karny, Thysanoptera: Thripidae) is an invasive insect pest of vegetables, legumes, and ornamentals besides being vector to several viruses. It poses a threat to domestic and international plant biosecurity and can invade and establish in new areas. Here, we report a polymerase spiral reaction (PSR)-based isothermal assay for rapid, sensitive, specific, low-cost, and on-site detection of *T. palmi*. To the best of our knowledge, this is the first application of PSR in the identification of any insect species. A primer pair designed based on 3′-polymorphism of mtCOIII region can specifically identify *T. palmi* without any cross-reactivity with predominant thrips species. The assay uses crude lysate of a single thrips saving time and reagents involved in nucleic acid extraction. The presence of *T. palmi* is visualized by the appearance of bright fluorescence under ultraviolet light or a change in reaction color thus avoiding gel electrophoresis steps. The entire process can be completed in 70 min on-site using only an ordinary water bath. The assay is sensitive to detecting as little as 50 attograms of *T. palmi* template. The assay was validated with known thrips specimens and found to be efficient in diagnosing *T. palmi* under natural conditions. The described method will be useful for non-expert personnel to detect an early infestation, accidental introduction to a new area, restrict the spread of diseases and formulate appropriate management strategies.

## Introduction

Thrips are minute, slender, fringed-wing insects that cause considerable damage by puncturing plant tissues and sucking cell content. Besides direct damages, they transmit several tospo-, illar-, sobemo-, machlomo-, and carmoviruses (Ghosh et al., 2021a). About 6,353 extant thrips species in 785 genera are known (ThripsWiki, 2022). Their small size and cryptic habit render the diagnosis of thrips species very challenging. Melon thrips, *Thrips palmi* Karny (Thysanoptera, Thripidae) is one of the predominant thrips species infesting crop plants of more than 20 plant families including Asteraceae, Cucurbitaceae, Leguminosae, Malvaceae, and Solanaceae (Nakahara, 1984; Talekar, 1991). This thrips was restricted to southeast Asia until 1980 (Bhatti, 1980; Hirose, 1991) and has since spread across Asia and was introduced into Africa, Australia, South America, Hawaii, the Caribbean, and Florida post-1990s (Smith et al., 1997; Bournier, 1999; MacLeod et al., 2004). Outbreaks of *T. palmi* in the Netherlands (1988–98), United Kingdom (2000–2001), England (2000), Portugal (2004), and Germany (2014) (EPPPO, 2021) were followed by intensified eradication programmes over two complete cropping cycles. In the EPPO region, *T. palmi* poses a serious threat to a wide variety of glasshouse and field crops and is listed as an A1 quarantine pest (EPPPO, 2021). Over 80% of the watermelon crops were destroyed by *T. palmi* in the Philippines (Medina 1980). Plantings of aubergine had to be abandoned due to the heavy infestation of *T. palmi* (Bernardo, 1991). *T. palmi* has had disastrous economic effects on cucurbits (melons, cucumbers) and solanaceous crops (aubergines, capsicum). It is also a major pest of potatoes, cotton, tobacco, beans, yellow squash, and ornamentals, and feeds on several weed species (Miyazaki et al., 1984; Bournier, 1986; Johnson 1986; Guyot 1988; Cooper, 1991; Ghosh et al., 2020). Infestation in foliage leads to bronzing and premature shedding. The fruits get deformed due to heavy infestation resulting in lowered market value (Seal and Sabines, 2012). Besides direct damage caused by feeding, *T. palmi* can transmit seven tospoviruses (Rotenberg et al., 2015; Ghosh et al., 2017, 2019; Jagdale and Ghosh, 2019; Ghosh et al., 2021b) including capsicum chlorosis virus (CaCV), groundnut bud necrosis virus (GBNV), watermelon bud necrosis virus (WBNV), and watermelon silver mottle virus (WSMoV). *T. palmi*-transmitted GBNV alone causes an annual economic loss of over US$ 89 million in Asia (Singh and Krishna reddy 1996). Yield losses of around 90 and 29% have been reported in peanut and potato, respectively due to GBNV infection in India (Singh and Srivastava, 1995; Singh et al., 1997). A 39–100% infection of WBNV and yield losses of up to 100% was reported in watermelon (Krishna reddy and Singh, 1993; Jain et al., 1998).

Early and accurate identification is the key to an adequate response to plant health threats and minimizing the risk of outbreaks of regulated and other harmful thrips species. As *T. palmi* is difficult to detect at low density on crops and in consignments, sensitive and rapid detection methods are imperative. The conventional morphological key-based identification of thrips is time-consuming, adult stage-specific, and demands expert knowledge. In recent times, the application of molecular biology tools in thrips diagnosis has helped overcome the limitations of morphological character-based identification (Ghosh et al., 2021a). The molecular assays such as polymerase chain reaction (PCR) (Jangra et al., 2020a; Ghosh et al., 2020), random amplification of polymorphic DNA (RAPD) (Mainali et al., 2008), restriction fragment length polymorphism (RFLP) (Rugman-Jones et al., 2006), simple sequence repeats (SSRs) (Cao et al., 2019), and real-time PCR (Przybylska et al., 2018) for diagnosis of *T. palmi* but these molecular methods have limited utility for point-of-need applications. The assays also suffer resource-limited settings and are not portable. Isothermal amplification-based assays like loop-mediated isothermal amplification (LAMP) (Przybylska et al., 2015) and recombinase polymerase amplification (RPA) (Priti et al., 2021) for rapid identification of *T. palmi* are advantageous in this context. LAMP and RPA do not require any sophisticated laboratory equipment and can be performed at the field level within a short time. However, the designing of primers for LAMP assay is complicated with a high likelihood of non-specific amplification. RPA reagents are neither cost-effective nor readily available. The objective of the present study was to develop a rapid, cost-effective, and user-friendly assay for the on-site diagnosis of thrips species with a limited setup. We report here an isothermal, on-site assay for identification of *T. palmi* based on polymerase spiral reaction (PSR) which utilizes a single set of primers with adapter oligonucleotide sequences derived from an exogenous gene for isothermal amplification of nucleic acids (Liu et al., 2015). It does not require any sophisticated equipment, and reagents are readily available. PSR has been successfully implemented in clinical settings and food testing (Liu et al., 2015; Gupta et al., 2017; Malla et al., 2018; Ji et al., 2019; Milton et al., 2020; Sharma et al., 2020, 2022; Tomar et al., 2020; Maiti et al., 2022). This is the first experimental demonstration where PSR has been utilized for the detection of insect species. The assay can be executed using only a water bath and the results can be seen with the naked eye. The assay reported here is relatively simple, sensitive, fast, and easy to use and can be optimized for other invasive insects. The outcome of the study aims to minimize crop losses by early detection, reducing the risk of outbreaks, quarantining alien invasion, and adopting adequate pest management strategies.

## Materials and Methods

### Establishment of a Homogenous *Thrips palmi* Population

An isofemale population of *T. palmi* maintained at Advanced Centre for Plant Virology, Indian Agricultural Research Institute (IARI), New Delhi since 2018 was used in this study. The population was generated from a single adult female on eggplant (var. Navkiran, Mahyco, Jalna, India) under controlled environmental conditions. The population was identified based on morphometric keys (Bhatti 1980; Cluever and Smith, 2017) and confirmed by mitochondrial subunit I (mtCOI) sequencing and *T. palmi*-specific PCR (Jangra et al., 2020a). Adults of *T. palmi* were collected from the stock population using a fine Camel hairbrush (Kokuyo Camlin Ltd., Mumbai, India) and used in this study.

### DNA Isolation From Thrips

The assay was initially performed using purified DNA and later optimized with crude lysate of thrips. Total genomic DNA was isolated from a single thrips adult using DNeasy Blood and Tissue Kit (Qiagen, Hilden, Germany) with modifications. Briefly, the insect was crushed in 180 µl of ATL buffer with a sterile micro-pestle (Dewsil Scientific Pvt. Ltd., New Delhi, India) and the lysate was incubated at 56°C for 1 h. The incubation was followed by the addition of 200 µl of AL buffer and 200 µl of molecular grade ethanol (Merck, Darmstadt, Germany). This was followed by washing with 500 µl wash buffers AW1 and AW2, respectively. After washing, the DNA was eluted in 20 µl of sterile water (Puregene, Genetix Biotech Asia Pvt. Ltd, New Delhi, India) and stored at −20°C until further use.

### Design of Polymerase Spiral Reaction Primers

The PSR primer pairs used in this study were designed as described by Liu et al. (2015). A total of 44 inter-transcribed spacer 2 (ITS2), 244 mitochondrial cytochrome oxidase subunit I (mtCOI), and 25 mtCOIII sequences of *T. palmi* available in NCBI were aligned using Clustal W in MEGA-X software package (Kumar et al., 2018) and conserved regions were identified. The forward and reverse primers for the above-mentioned regions were analyzed using Oligo Analyzer Tool in the IDT database (http://eu.idtdna.com/calc/analyzer) to identify possible 3ʹ-self-complimentary or hairpin structures. Further, an adapter oligonucleotide sequence of exogenous origin was added to the 5ʹ-end of the primers in such a manner that the melting temperature (Tm) of the adapter sequence was 5°C lower than the primer sequence. The adapter sequence of forward primers was exactly reverse to the adapter sequence in reverse primers. A total of four primer pairs were designed, two from ITS2 region and one each from mtCOI and mtCOIII regions (Table 1). The length of the primers was kept between 20–23 bp excluding the adaptor sequence. There was no mismatch at the 3′-end of the primers and ≤ 3 mismatches in the entire primer sequence. The specificity of the primer pairs was confirmed using Primer-BLAST.

**TABLE 1:** List of primers used for PSR assay of *T. palmi*.

Sl. No	Forward primer				Reverse primer				Target gene	Amplicon size (bp)
	Name	Sequence (5ʹ-3ʹ)	Tm (°C)	Length (nt)	Name	Sequence (5ʹ-3ʹ)	Tm (°C)	Length (nt)		
1	AG329F	acg​att​cgt​aca​tag​aag​tat​agT​GGC​TGC​TGA​ACC​GCT​CCG	67.0	42	AG330R	gat​atg​aag​ata​cat​gct​tag​caG​TGA​ATC​GGA​GCG​AGG​AGG​C	66.2	43	ITS2	180
2	AG337F	acg​att​cgt​aca​tag​aag​tat​agT​CCC​GAT​ATA​GCA​TTT​CCA​CGA	63.8	45	AG338R	gat​atg​aag​ata​cat​gct​tag​caG​AGG​ATA​CCC​CAG​CTA​AAT​GGA	64.5	45	mtCOI	200
3	AG339F	acg​att​cgt​aca​tag​aag​tat​agG​AGT​GAC​ATT​AAC​AGC​AGC​TCA	64.1	45	AG340R	gat​atg​aag​ata​cat​gct​tag​caG​AATACCATGGAATCCTGT	63.6	46	mtCOIII	200
4	AG341F	acg​att​cgt​aca​tag​aag​tat​agG​TTG​CGA​TGT​GTT​TCT​GCA​C	64.3	43	AG342R	gat​atg​aag​ata​cat​gct​tag​caA​ATA​CAA​CAT​CGA​GGT​GCC​C	63.8	43	ITS2	175

Nucleotide sequence in lowercase at 5′ indicates adapter sequence. Underline corresponds to NcoI enzyme restriction site

### Validation of Polymerase Spiral Reaction Primers

PSR primer pairs were first validated in a gradient PCR. The 25 μl PCR reaction comprised of 50 ng DNA template, 1X DreamTaq buffer (Thermo Fisher Scientific, Massachusetts, United States), 0.4 µM each forward and reverse primer (Integrated DNA Technologies, Iowa, United States), 260 µM dNTP mix (Thermo Fisher Scientific), and 2 U DreamTaq DNA Polymerase (Thermo Fisher Scientific). PCR was carried out in a T100 Thermal Cycler (Bio-Rad, California, United States) at the following reaction conditions: 94°C for 3 min, 35 cycles of 94°C for 30 s, annealing at 60–65°C depending upon the primer pairs for 50 s, 72°C for 50 s, and a final extension at 72°C for 10 min. PCR products were resolved on 2% agarose gel (Lonza, Rockland, United States) stained with GoodView (BR Biochem, New Delhi, India) and visualized in a gel documentation system (MaestroGen Inc, Hsinchu City, Taiwan) with a 1 kb plus DNA ladder (Thermo Fisher Scientific).

### Optimization of Polymerase Spiral Reaction Assay

The PSR assay was done using a temperature gradient of 60–69°C and reaction time of 60–90 min. The primer concentration was also optimized. The reaction mixture comprised of 2.5 µl of 10X Thermopol reaction buffer (New England Biolabs, Massachusetts, United States) containing 20 mM Tris-HCl, 10 mM KCl, 10 mM (NH_4_)_2_SO_4_, 2 mM MgSO_4_, and 0.1% Tween 20, 10–40 µM each forward and reverse primer, 1.4 mM dNTP mix (Thermo Fisher Scientific), 0.8 M Betaine (Merck), 6 mM MgSO_4_ (New England Biolabs), 8–16 U of *Bst* DNA polymerase large fragment (New England Biolabs), 50 ng DNA template, and the final volume was adjusted to 25 µl with sterile distilled water. No-template water control (NTC) was used with each run.

Restriction digestion of PSR products was done using 5 µl of PSR product, 2 µl of *Nco*I FastDigest enzyme (Thermo Fisher Scientific), 2 µl 10X FastDigest Green Buffer (Thermo Fisher Scientific), in a final volume of 20 µl for 1 h at 37°C. The digested products were resolved in 2% agarose gel electrophoresis as described above. Based on the results of PSR, primer pair AG339F-AG340R was further assessed for cross-reactivity and sensitivity.

### Assessment of Cross-Reactivity

Potential cross-reactivity of the PSR primers was first assessed in conventional PCR and then in PSR. The primer pair AG339F-AG340R was assessed for cross-reactivity with the other congeneric and predominant thrips vectors viz. *T. tabaci*, *Scirtothrips dorsalis*, and *Frankliniella schultzei*. Isofemale populations of *T. tabaci* (GenBank accession no. MN594551)*, S. dorsalis* (accession no. OK398217)*,* and *F. schultzei* (accession no. MN594552) maintained at Advanced Centre for Plant Virology, IARI, New Delhi were used in the study. PCR was done in a 25 µl reaction mixture as described above with DNA templates from *T. palmi*, *T. tabaci*, *S. dorsalis*, and *F. schultzei*. PCR amplicons were resolved on 2% agarose gel (Lonza) as described above.

The specificity of the primer pairs was further confirmed in PSR. PSR assay was performed in a 25 µl reaction mixture with the DNA templates from *T. palmi*, *T. tabaci*, *S. dorsalis*, and *F. schultzei*. The amplified products were digested by *Nco*I FastDigest enzyme. The amplified and digested PSR products were resolved on 2% agarose gel as described above.

### Sensitivity of Polymerase Spiral Reaction Assay

The sensitivity of the PSR assay using primer pair AG339F-AG340R was determined using a 10-fold serial dilution of template DNA. The initial DNA concentration of 50 ng/μl was serially diluted up to 5 ng/μl × 10^−8^ ng/μl and was used in PSR assays as described above. The amplified PSR products were resolved on 2% agarose. Further, the sensitivity of the PSR assay was compared with PCR using the same dilutions of template DNA. PCR was carried out in a 25 µl reaction mixture as above and products were resolved on 2% agarose gel.

### On-Site Polymerase Spiral Reaction Assay Using Crude Thrips Lysate

For on-site detection, DNA extraction steps were eliminated by using a crude lysate of thrips. A single *T. palmi* was collected in a 1.5 ml microcentrifuge tube (Tarsons, Kolkata, India) and 20 µl of sterile water (Genetix Biotech Asia Pvt. Ltd.) was added. The specimen was crushed within the tube with the help of a micro-pestle (Dewsil Scientific Pvt. Ltd.). The tube was placed in a water bath (Jaibro, New Delhi, India) at 100°C for 2 min. The lysate so obtained was directly used for amplification in PSR as described above.

### Optimization of Visual Detection Using Colorimetric and Fluorescent Dyes

To simplify the end-point detection and make the PSR assay portable, the gel electrophoresis step was eliminated by using DNA intercalating fluorescent and colorimetric dyes. After completion of the PSR reaction, 1 µl of SYBR Green I (Thermo Fischer Scientific) was added to each reaction. To make the visual detection more cost-effective, SYBR Green I was replaced with GoodView (BR Biochem). Similar to SYBR Green I, 1 µl of GoodView was added to each tube after the completion of the reaction. The presence or absence of fluorescence was detected under ultraviolet (UV) light. End-point detection was further simplified using a colorimetric dye. Two micro liter of 3 mM hydroxy naphthol blue (HNB, Sisco Research Laboratories Pvt. Ltd, Mumbai, India) was added at the start of the reaction and mixed well by pipetting. PSR was done by incubation at 65°C for 60 min in a water bath. A change in reaction color indicated the presence of *T. palmi*.

### Validation of Polymerase Spiral Reaction From Field Samples

The PSR assay was validated using known thrips species. Further, the assay was used to discriminate *T. palmi* from randomly collected field specimens. Thrips specimens were collected from eggplant, capsicum, onion, tomato, okra, mungbean, cowpea, cucumber, periwinkle, and ridge gourd at experimental fields of IARI. Thrips were collected from both leaves and flowers. Specimens were packed in sealed sample bags and carried to the laboratory. Crude lysate was extracted from single thrips individuals and PSR was done as described above. The presence or absence of *T. palmi* was confirmed by the GoodView fluorescence using a UV torch and/or change in color while using HNB. MtCOI region of representative field specimens was sequenced to substantiate the specificity of the PSR assay.

## Results

### Homogenous *Thrips palmi* Population

A homogeneous population developed from a single adult female of *T. palmi* was used in this study. The adults were yellowish in color. The quadrangular head had three brick red ocelli in a triangular formation. A pair of setae were located outside this ocellar triangle. The antennae had seven segments. The females had sharp ovipositors at the apex of abdomen, whereas the apex of males was round and blunt. Males were slightly smaller than females and faster in their movement.

PCR using *T. palmi*-specific primers for ITS2 region (Jangra et al., 2020a) yielded the expected amplicon of 568 bp on an agarose gel (accession number MN194202). Further, the nucleotide sequence of a 660 bp PCR amplified mtCOI product using primer pair LCO1490 and HCO2198 (Folmer et al., 1994) showed 100% sequence identity with *T. palmi*. The sequence can be retrieved from NCBI with accession number OK398218.

### Designing and Validation of Polymerase Spiral Reaction Primers

Four pairs of primers viz. AG329F-AG330R, AG341F-AG342R, AG337F-AG338R and AG339F-AG340R were designed based on sequence polymorphism in mtCOI, mtCOIII, and ITS2 regions. The length and GC content of the primer pairs ranged from 43–46 nt and 40–68.4%, respectively (Table 1). There was no mismatch at the 3′-end of the primers. The sequences of primer pair, AG329F-AG330R were highly conserved without any mismatch among 44 *T. palmi* ITS2 sequences used to design the primers. The rest of the primer sequences had ≤ 3 mismatches across the entire primer length. The melting temperature of all the primer pairs ranged between 63.6–67°C. Primer-BLAST analysis showed a low probability for secondary structure formation, hairpin loop formation, and 3ʹ-self complementarity. The primer pairs were found to be specific to *T. palmi* and intra-specific variations of *T. palmi* could be amplified. Primer-BLAST analysis of the primer pairs, AG329F-AG330R, AG337F-AG338R, AG339F-AG340R, and AG341F-AG342R predicated amplicon sizes of 179, 198, 198, and 175 bp, respectively without any cross-reactivity to sequences of other thrips species.

In gradient PCR at 60–65°C, primer pair AG329F-AG330R yielded a ∼180 bp amplicon of ITS2 at annealing temperatures of 62–65.3°C. Sharp DNA bands of ∼200 bp were observed in PCR with primer pairs AG337F-AG338R and AG339F-AG340R at all tested annealing temperatures. Similarly, a distinct band of ∼175 bp was observed with primer pair, AG341F-AG342R. No amplification was observed in NTC. The amplified products were sequenced to confirm the specificity of the PCR reactions. The sequences can be retrieved with the GenBank accession no. provided in the data availability statement.

### Optimization of Polymerase Spiral Reaction Conditions

Among the four pairs of primers tested in gradient PCR, the primer pair AG339F-AG340R could only amplify the DNA template of *T. palmi* in PSR satisfactorily. The PSR assay was optimized for primer concentration, polymerase concentration, and reaction temperature. Primer concentration of 40 µM produced the best amplification and hence was adopted throughout the assay. A reaction temperature range of 60–69°C was assessed, however, the best amplification was observed at 65°C. Further, the concentration of *Bst* DNA polymerase was standardized at 16 U per reaction. The optimized PSR reaction mixture was comprised of 2.5 µl of 10X Thermopol reaction buffer, 40 µM each forward and reverse primer, 1.4 mM dNTP mix, 0.8 M Betaine, 12 mM MgSO_4,_ 16 U of *Bst* DNA polymerase large fragment, 50 ng DNA template, and the final volume was adjusted to 25 µl with sterile distilled water. The incubation time of PSR at 65°C was assessed for 60–90 min. However, we did not observe any significant difference in amplification between 60 and 90 min, hence a reaction time of 60 min was adopted.

**FIGURE 1:**
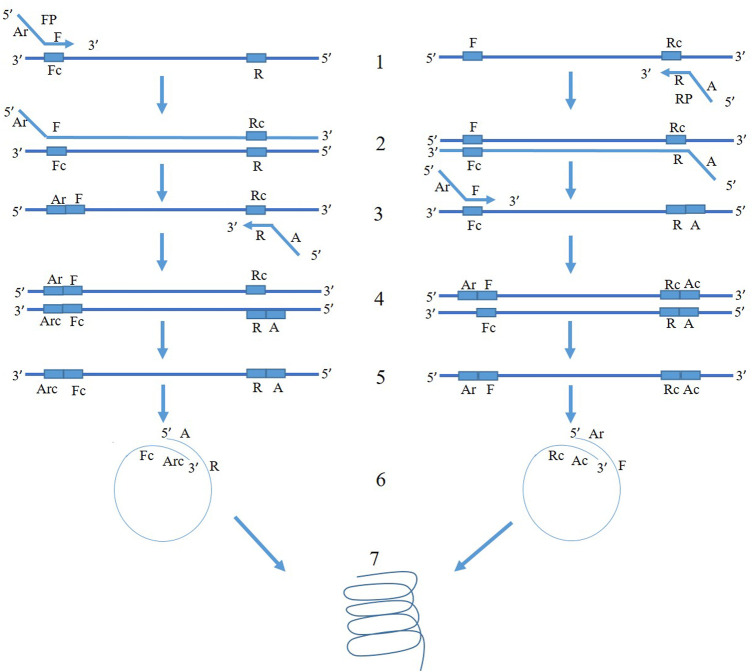
Schematic representation of PSR assay. FP and RP denote forward and reverse primers targeting the mtCOIII region of *T. palmi*. The 3′ sequence of forward primer is denoted as “F” and that of reverse primer is denoted as “R” and is complementary to the target mtCOIII sequence. An adapter sequence (A) was added at 5′ of revere primer. The adapter sequence of forward primer (Ar) is reverse to adapter sequence of reverse primer (A). At 65°C, the double-stranded template DNA unfolds in presence of Betaine. In the left panel, F segment of the forward primer (FP) anneals to the complementary single-strand of DNA (step 1) and extends (step 2). After the melting, the R segment of the reverse primer (RP) binds to it (step 3) and extends (step 4). Now, both the strands melt and form a single chain (step 5). As the sequences of A and Arc are reverse complementary to each other, it makes a circular structure in step 6. The 3′ end continues to extend and gives spiral amplification (step 7). Similarly, mechanism of amplification happens for another single-stranded chain in the right panel.

**FIGURE 2:**
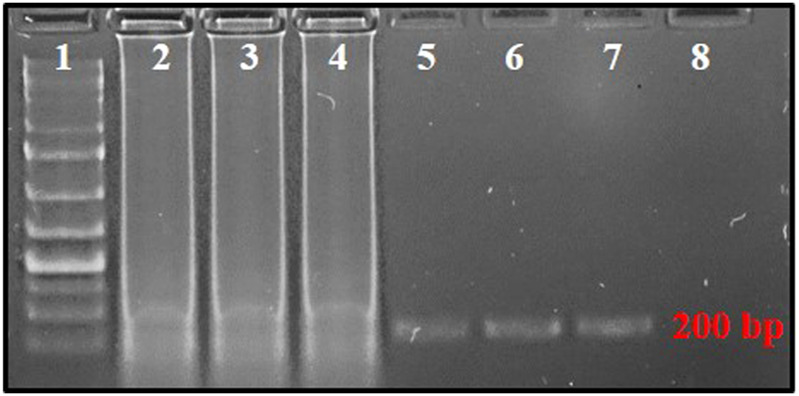
PSR-amplified products using primer pair AG339F-AG340R resolved on 2% agarose gel. Lane 1: 1 kb plus DNA ladder, lanes 2–4: PSR amplicons with *T. palmi* DNA templates, lanes 5–7: *Nco*I-digested PSR amplicons, 8: no-template water control.

The mechanism of PSR is illustrated in Figure 1. At 65°C, the double-stranded template DNA unfolds in presence of Betaine. After the melting, the F segment of the forward primer (FP) and R segment of the reverse primer (RP) anneal to the complementary single-strands of the DNA and extend (Figure 1). Both the strands melt and form a single chain. As the sequences of A-Arc and Ar-Ac are reverse complementary to each other, they make circular structures and extend to generate a spiral amplification in presence of *Bst* DNA polymerase. PSR amplicons were subjected to 2% agarose gel electrophoresis and multiple bands within a smear of DNA were observed. No such amplification was observed in NTC. The *Nco*I-digested PSR product yielded a single, strong band of ∼200 bp in 2% agarose gel electrophoresis (Figure 2).

### Assessment of Cross-Reactivity With Other Predominant Thrips Species

**FIGURE 3:**
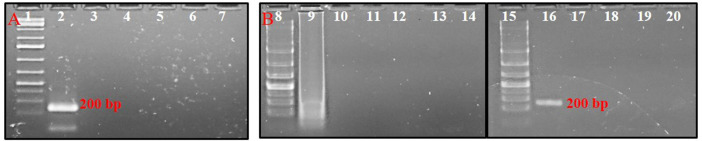
Assessment of cross-reactivity of primer pair AG339F-AG340R in **(A)**. PCR and **(B)**. PSR. Lanes 1, 8, 15: 1 kb plus DNA ladder, lanes 2–6: PCR with DNA templates of *T. palmi* (2), *S. dorsalis* (3), *T. tabaci* (3), and *F. schultzei* (4), respectively. Lanes 9–13: PSR with DNA templates of *T. palmi* (9), *S. dorsalis* (10), *T. tabaci* (11), and *F. schultzei* (12), respectively. Lanes 16–19: *Nco*I-digested PSR amplicons from DNA templates of *T. palmi* (16), *S. dorsalis* (17), *T. tabaci* (18), and *F. schultzei* (19), respectively. Lanes 7, 14, 20: no-template water control.

No cross-reactivity with other tested thrips species was observed in either conventional PCR or PSR. The primer pair, AG339F-AG340R showed no cross-reactivity with DNA templates from predominant thrips vectors viz. *T. tabaci*, *S. dorsalis*, and *F. schultzei*. The PCR with primer pair AG339F-AG340R yielded an amplicon of ∼200 bp in DNA template from *T. palmi*, while no amplification was observed in DNA template from *T. tabaci*, *S. dorsalis*, and *F. schultzei* and NTC (Figure 3A). Similarly, PSR amplification was observed only with DNA template from *T. palmi*, but not for *T. tabaci*, *S. dorsalis*, *F. schultzei,* and NTC (Figure 3B).

### Sensitivity of Polymerase Spiral Reaction Assay

**FIGURE 4:**
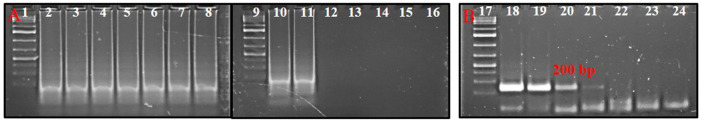
**(A)** Sensitivity of PSR assay using primer pair AG339F-AG340R. Ten-fold serially-diluted *T. palmi* DNA was used as a template and products resolved on 2% agarose gel. Lanes 1, 9, 17: 1 kb plus DNA ladder. Lanes 2–8, 10–15: PSR amplicons of serially-diluted *T. palmi* template of 5 ng × 10 ng (2), 5 ng (3), 5 ng × 10^−1^ ng (4), 5 ng × 10^−2^ ng (5), 5 ng × 10^−3^ ng (6), 5 ng × 10^−4^ ng (7), 5 ng × 10^−5^ ng (8), 5 ng × 10^−6^ ng (10), 5 ng × 10^−7^ ng (11), 5 ng × 10^−8^ ng (12), 5 ng × 10^−9^ ng (13), 5 ng × 10^−10^ ng (14), 5 ng × 10^−11^ ng (15), lane 16: no-template water control. **(B)**. Sensitivity of PCR assessed using the same primer pair and template. Lane 17: 1 kb plus DNA ladder, lanes 18–23: PCR amplicons of serially diluted *T. palmi* template of 5 ng × 10 ng (18), 5 ng (19), 5 ng × 10^−1^ ng (20), 5 ng × 10^−2^ ng (21), 5 ng × 10^−3^ ng (22), 5 ng × 10^−4^ ng (23), lane 24: no-template water control.

The serially diluted *T. palmi* DNA templates (5 ng × 10^1^-10^−8^ ng) were subjected to PSR for determining the sensitivity of the assay. PSR with primer pair AG339F-AG340R showed amplification corresponding to a DNA concentration up to 5 ng × 10^−7^ ng, while no amplification was observed in DNA concentration of 5 ng × 10^−8^ ng and NTC (Figure 4A). The sensitivity of the PSR assay was compared with conventional PCR. PCR could amplify a ∼200 bp product for template DNA concentration up to 5 ng × 10^−2^ ng (Figure 4B). The results indicated that PSR assay was 10^5^ times more sensitive than conventional PCR and could detect as low as 50 atto-grams of template DNA (Figure 4).

### Polymerase Spiral Reaction-Based On-Site Detection of Thrips

**FIGURE 5:**
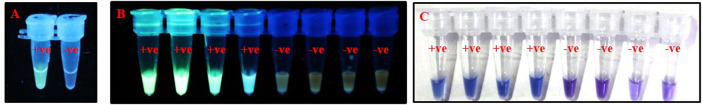
Visualization of PSR products using **(A)**. SYBR Green I, **(B)**. GoodView, and **(C)**. HNB. PSR was done using crude lysate of *T. palmi* as positive (+ve) and water as negative (-ve) control. Addition of 1 µl of SYBR Green I and GoodView after completion of PSR reaction showed fluorescence under UV light in positive samples, whereas no fluorescence was observed in negative samples. The PSR reaction was mixed with 2 µl of 3 mM HNB prior to amplification and showed a change in color from violet to sky blue in the case of *T. palmi*, while no corresponding color change was observed in negative samples.

The time required for isolation of DNA for PSR was minimized by using a crude lysate of thrips. The crude lysate was prepared by crushing the thrips in sterile water and incubating at 100°C for 2 min in a water bath. PSR could amplify the expected product from crude lysate as efficiently as from purified DNA templates (data not shown). Furthermore, crude extracts from thrips could be made on-site and did not require any laboratory equipment that would be needed for CTAB or kit-based DNA extraction. PSR assay with crude thrips lysate showed multiple bands within a smear like PSR with a purified DNA template. Further, to make the assay even more simple and rapid, the need for agarose gel electrophoresis was eliminated by using fluorescent and colorimetric dyes. SYBR Green I and/or GoodView were added to the reaction tubes after completion of the assay. The PSR assay with crude lysate of *T. palmi* emitted bright fluorescence under UV light while no fluorescence was observed in negative samples (Figures 5A,B). In colorimetric end-point detection, HNB was added to the reaction mixture at the beginning. A change in reaction color from violet to sky blue was recorded in the PSR assay with *T. palmi* lysate, whereas no such color change was observed in negative samples (Figure 5C). The entire process could be completed in around 70 min.

### Validation of Polymerase Spiral Reaction Assay and Identification of Thrips From Natural Vegetation

**FIGURE 6:**
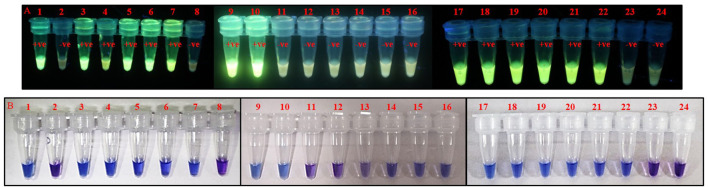
Identification of *T. palmi* collected from different crop plants using PSR. Crude lysate of thrips was used in PSR with primer pair AG339F-AG340R. The presence of *T. palmi* was visualized using **(A)**. GoodView, and **(B)**. HNB. Tubes 1,9,17: positive control (*T. palmi*), tubes 2–7, 10–15, 18–23: unknown thrips collected from chili (2), mungbean (3, 21), ridge gourd-leaf (4, 22), okra (5, 20), cowpea (6, 19), eggplant (7, 10, 18), ridge gourd-flower (11), onion (12, 23), tomato (13), periwinkle (14, 15), tubes 8, 16, 24: no-template water control.

The PSR assay was performed rigorously and validated with more than 50 known thrips specimens of *T. palmi*, *S. dorsalis*, *T. tabaci*, and *F. schultzei*. This confirmed the reliability and reproducibility of the assay. Further, PSR was used to identify *T. palmi* on-site from randomly collected thrips specimens. PSR was done in a water bath with crude lysate of thrips using GoodView as well as HNB-based visual detection. Thrips populations were collected randomly from 14 crops. The presence of *T. palmi* was confirmed in eggplant, cowpea, cucumber, mungbean, okra, and ridge gourd (leaf) by the appearance of distinct fluorescence when the reaction tubes were exposed to UV light and/or change of reaction color from violet to sky blue (Figure 6). The thrips species collected from chilli, onion, tomato, ridge gourd (flower), and periwinkle were not *T. palmi* as indicated by the PSR assay results. MtCOI sequences of random positive and negative specimens (14 samples) further confirmed the specificity of the assay. The sequences can be retrieved from NCBI using GenBank accession no. provided in the data availability statement.

## Discussion

*T. palmi* is known to infest more than 200 plant species including important crops such as beans, cucumber, eggplant, gourds, groundnut, melons, peppers, potatoes, tomatoes, and ornamentals (MacLeod et al., 2004; Jangra et al., 2020b; Dhall et al., 2021; Mahanta et al., 2022). Under changing climatic conditions, outbreaks of *T. palmi* have become more frequent and severe (Stuart et al., 2011; Seal et al., 2013; Hong et al., 2019; Ghosh et al., 2020), posing a threat to international biosecurity. It can invade, establish, and expand into new areas. Being cryptic, it is always difficult to identify particular thrips species in large consignments. Moreover, the conventional identification of thrips at species level using morphological characters is limited to adult specimens. Identification of eggs and immature stages using morphological keys is not feasible as no reliable keys have been developed for immature stages. Eggs of *T. palmi* are inserted into the plant tissues, therefore, cannot be detected by conventional sampling and scouting techniques. The introduction of various molecular tools for the identification of insect species has made the process fast, sensitive, robust, and easier for non-expert personnel. The first PCR-based characterization of thrips species was undertaken during the late 1990s (Crespi et al., 1998). Subsequently, RAPD, RFLP, AFLP, SSR, SCAR, and qPCR have been reported for discrimination of thrips species (Fang et al., 2005; Meena et al., 2005; XiangQin et al., 2010; Przybylska et al., 2016; Przybylska et al., 2018; Cao et al., 2019; Ghosh et al., 2021a). Besides, the molecular techniques aid in identifying cryptic diversity, biotypes, host races, genetic structure, gene flow, reproductive isolation, and resolving species ambiguities in thrips which are otherwise impossible using morphological keys (Ghosh et al., 2021a). However, none of the above molecular techniques can be easily implemented on-site as they require sophisticated instruments for nucleic acid isolation, thermal cycling, and gel electrophoresis. That is the reason behind the low adoption of these techniques for field-based identification by scouting or quarantine personnel. Isothermal nucleic acid amplification methods are gaining popularity in rapid diagnostics for invasive species at the point of entry. Isothermal techniques like LAMP, helicase-dependent amplification (HDA), RPA serve as alternatives to PCR (Notomi et al., 2000; Blaser et al., 2018; Andreason et al., 2020; Priti et al., 2021). A LAMP assay developed for *T. palmi* works well for on-site detection (Przybylska et al., 2015). Recently, we reported an RPA-based assay for on-site identification of *T. palmi* that uses recombinase, single-stranded DNA binding protein, and strand displacing polymerase to amplify the target DNA at isothermal conditions (Priti et al., 2021).

The present study reports an alternative isothermal assay for on-site, rapid identification of thrips species. PSR is a unique amalgamation of isothermal LAMP and conventional PCR (Figure 1). Isothermal assay like LAMP uses more than four primers or a DNA helicase (HDA). PSR assay described in this study uses only one pair of primers and follows isothermal amplification. Designing PSR primers is also simpler like PCR primers. A stuffer oligo sequence (A, Ar) of exogenous origin was added on 5ʹ end of the primers (F, R). The melting temperature of the stuffer sequence (A and/or Ar) was set 5°C lower than the primer sequence (F, R) to ensure binding of primers to the target genes before the formation of “spiral structure” for amplification. In this study, the stuffer sequences were derived from an exogenous origin to avoid non-specific reactions. The PSR assay does not require a denaturation step, and the reaction starts as soon as the temperature reaches around 65°C.

PSR assay for clinical diagnosis of viral and bacterial diseases and food pathogens are highly specific to their respective target nucleic acid sequences (Liu et al., 2015; Gupta et al., 2017; Malla et al., 2018; Ji et al., 2019; Milton et al., 2020, 2021; Sharma et al., 2020, 2022; Tomar et al., 2020; Maiti et al., 2022). The PSR assay reported in the present study is specific to *T. palmi* mtCOIII region and does not cross-react to predominant thrips species. The mtCOIII is a potential marker for discrimination of thrips species because of larger interspecific distance and can amplify intraspecific variation (Glover et al., 2010; Ghosh et al., 2020; Jangra et al., 2020a). PSR assay could amplify as low as 50 atto-grams of template DNA of thrips and was 10^5^ times more sensitive than conventional PCR. The real-time PCR could amplify *T. palmi* up to 1 pg (Przybylska et al., 2018). The LAMP assay developed by Przybylska et al. (2015) could detect 2 × 10^−4^ of adult *T. palmi* DNA. The RPA assay for *T. palmi* had a detection threshold of 2 ng × 10^−10^ ng (Priti et al., 2021). The sensitivity of the PSR assay is higher than previously reported real-time PCR and LAMP and comparable to that of RPA. The advantages of PSR over RPA are the easy availability of enzymes and reagents and cost-effectiveness. We found that the cost-effectiveness ratio of RPA, LAMP, and PSR is around 5: 1.5: 1.

**FIGURE 7:**
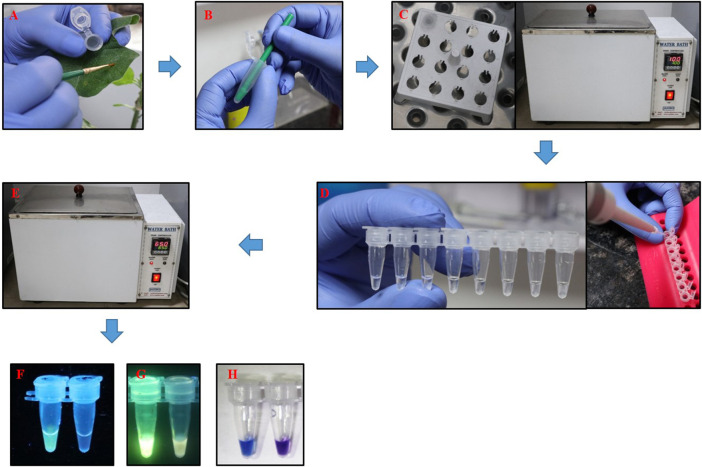
Workflow of PSR assay for rapid, on-site detection of *T. palmi*. **(A)**. Single adult *T. palmi* collected from a leaf using a Camel hairbrush and placed in a 1.5 mlmicrocentrifuge tube, **(B)**. Thrips crushed with the help of a micro-pestle in sterile distilled water, **(C)**. Micro-centrifuge tube placed in a floating rack and incubated at 100°C in a water bath for 2 min, **(D)**. PSR mixture prepared in 0.5 ml PCR tubes, **(E)**. PSR reaction incubated in a water bath at 65°C for 60 min. Visual detection of presence or absence of *T. palmi* using **(F)**. SYBR Green I, **(G)**. GoodView. Fluorescence was observed in the positive samples while no fluorescence was detected in negative samples, **(H)**. Visual detection of *T. palmi* PSR products using HNB. Change in color from violet to sky blue indicates the presence of *T. palmi*, while no color change was observed in negative samples.

The entire process of PSR assay could be completed in 70 min at 65°C without the use of any sophisticated laboratory equipment. Crude extraction from thrips could be completed in 5 min, preparation of PSR mixture took around 5 min, followed by 60 min incubation. A workflow of the PSR assay for the detection of *T. palmi* is illustrated in Figure 7. LAMP assay developed for identification of *T. palmi* could also be completed within the same time and temperature range. The advantages of PSR assay are in overcoming the difficulties of LAMP assay that requires designing of complicated primer pairs and PSR has a lower risk of non-specific amplification (Sahoo et al., 2016; Zou et al., 2020). PSR utilizes the same *Bst* polymerase as LAMP but needs only one pair of primers with an exogenous adapter sequence for spiral isothermal amplification.

The PSR assay was further simplified by eliminating the need for gel electrophoresis. The presence of *T. palmi* could be detected by the appearance of distinct fluorescence under UV light. Further, the need for a UV light was eliminated by using colorimetric HNB instead. The presence of *T. palmi* was detected by a change in dye color from violet to sky blue. HNB is an indicator of metal ions, reported as a quantitative chemical indicator for Mg^2+^ ions (Ito and Ueno, 1970). It has been successfully employed by several researchers in LAMP and RPA assays (Priti et al., 2021; Wang et al., 2021). The change in reaction color from violet (negative) to sky blue (positive) in PSR was induced by the chelation of Mg^2+^ ions by dNTPs (Gelfand, 1989). The use of HNB for visual detection offers advantages over other diagnostics tools as the results can be viewed by the naked eye without opening the tubes. The assay was validated over a large number of known specimens and tested randomly on collected thrips populations from natural vegetation. This indicated the specificity, reliability, and reproducibility of the assay.

The availability of several molecular methods will strengthen the on-site, rapid detection of thrips species. The on-site PSR-based detection for *T. palmi* reported in the study is fast, sensitive, specific, user-friendly, and affordable. This would be a better alternative to presently available molecular tools for on-site diagnosis. To the best of our knowledge, this is the first application of PSR in the identification of any insect species. The detection assay can be performed by non-expert personnel at the field level without any sophisticated laboratory equipment. The assay will strengthen the biosecurity infrastructure and decision support system for insect pest management. Rapid and early detection will help prevent outbreaks of *T. palmi* in endemic areas and restrict its introduction or spread into new areas. Similar assays can also be developed for the diagnosis of other insect species of interest.

## Data Availability

The datasets generated and/or analyzed during the current study are available in the NCBI database and can be accessed using the accession numbers MN194202, MN594551, MN594552, OL311798, OK398217, OK398218, OK342116, OK326735, OK626595, OK626652, OK631726, OK626698, OK626700, OK631711, OK626779, OK626783, OK626787, and OK641925.
